# {2,2′-[(2,2-Dimethyl­propane-1,3-diyl­dinitrilo)­bis­(phenyl­methyl­idyne)]­diphenolato}nickel(II)

**DOI:** 10.1107/S1600536811029813

**Published:** 2011-08-02

**Authors:** Hadi Kargar, Reza Kia, Majid Moghadam, Fatemeh Froozandeh, Muhammad Nawaz Tahir

**Affiliations:** a Chemistry Department, Payame Noor University, Tehran 19395-4697, Iran; b X-ray Crystallography Laboratory, Plasma Physics Research Center, and Department of Chemistry, Science and Research Branch, Islamic Azad University, Tehran, Iran; c Catalysis Division, Department of Chemistry, University of Isfahan, Isfahan 81746-73441, Iran; d Department of Physics, University of Sargodha, Punjab, Pakistan

## Abstract

The asymmetric unit of the title complex, [Ni(C_31_H_28_N_2_O_2_)], comprises two crystallographically independent mol­ecules. The geometry around the Ni^II^ atom in each mol­ecule is distorted square planar. The dihedral angles between the two phen­oxy rings in each mol­ecule are 17.8 (4) and 36.5 (4)°. The crystal packing is stabilized by weak π–π inter­actions [centroid–centroid distance = 3.758 (5) Å] and C—H⋯π inter­actions.

## Related literature

For standard values of bond lengths, see: Allen *et al.* (1987 ▶). For background on tetra­dentate Schiff bases and their complexes, see: Kargar *et al.* (2010 ▶, 2009 ▶).
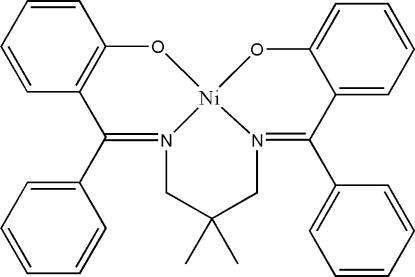


## Experimental

### 

#### Crystal data

[Ni(C_31_H_28_N_2_O_2_)]
*M* *_r_* = 519.26
Monoclinic, 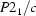

*a* = 23.722 (3) Å
*b* = 9.4716 (6) Å
*c* = 26.961 (4) Å
β = 124.319 (9)°
*V* = 5003.2 (10) Å^3^
*Z* = 8
Mo *K*α radiation
μ = 0.81 mm^−1^
*T* = 291 K
0.24 × 0.12 × 0.08 mm

#### Data collection

Stoe IPDS 2T image-plate diffractometer
Absorption correction: multi-scan [*MULABS* (Blessing, 1995 ▶) in *PLATON* (Spek, 2009 ▶)] *T*_min_ = 0.830, *T*_max_ = 1.000
23324 measured reflections
8608 independent reflections
2512 reflections with *I* > 2σ(*I*)
*R*_int_ = 0.117

#### Refinement

*R*[*F* ^2^ > 2σ(*F* ^2^)] = 0.053
*wR*(*F* ^2^) = 0.097
*S* = 0.61
8608 reflections
649 parameters
H-atom parameters constrained
Δρ_max_ = 0.23 e Å^−3^
Δρ_min_ = −0.24 e Å^−3^

### 

Data collection: *X-AREA* (Stoe & Cie, 2009 ▶); cell refinement: *X-AREA*; data reduction: *X-RED* (Stoe & Cie, 2009 ▶); program(s) used to solve structure: *SHELXS97* (Sheldrick, 2008 ▶); program(s) used to refine structure: *SHELXL97* (Sheldrick, 2008 ▶); molecular graphics: *SHELXTL* (Sheldrick, 2008 ▶); software used to prepare material for publication: *SHELXTL* and *PLATON* (Spek, 2009 ▶).

## Supplementary Material

Crystal structure: contains datablock(s) global, I. DOI: 10.1107/S1600536811029813/su2297sup1.cif

Structure factors: contains datablock(s) I. DOI: 10.1107/S1600536811029813/su2297Isup2.hkl

Additional supplementary materials:  crystallographic information; 3D view; checkCIF report

## Figures and Tables

**Table 1:** Table 1 ▶. C—H⋯π inter­actions (Å, °) *Cg*2, *Cg*3 and *Cg*4 are the centroids of the C18–C23, C32–C37 and C55–C60 rings, respectively.

C—H⋯*Cg*	C—H	H⋯*Cg*	C⋯*Cg*	C—H⋯*Cg*
C21—H21*A*⋯*Cg*2^ii^	0.93	2.90	3.757 (11)	153
C41—H41*A*⋯*Cg*3^iii^	0.93	2.83	3.680 (12)	153
C44—H44*A*⋯*Cg*4^iv^	0.93	2.95	3.708 (10)	149
C47—H47*A*⋯*Cg*4^v^	0.93	2.92	3.884 (9)	171

Symmetry codes: (ii) *x*, 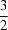 − *y*, 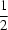 + *z*; (iii) 1 − *x*, 2 − *y*, 1 − *z*; (iv) 1 − *x*, −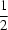 + *y*, 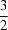 − *z*; (v) 1 − *x*, 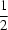 + *y*, 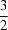 − *z*.

**Fig. 1.**
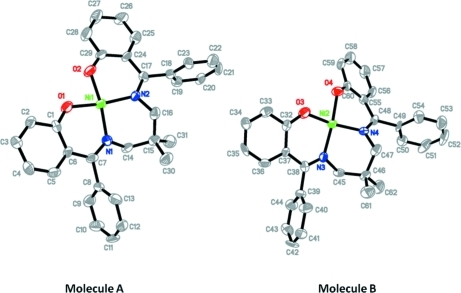
The molecular structure of the two independent molecules (A and B) of the title compound, showing 30% probability displacement ellipsoids and the atomic numbering [H-atoms have been removed for clarity].

## supplementary crystallographic information

### Comment

Schiff base ligands are one of the most prevalent systems in coordination chemistry. As part of a general study of potentially tetradenate Schiff bases and their complexes (Kargar *et al.*, 2009; Kargar *et al.*, 2010), we have determined the crystal structure of the title compound.

The asymmetric unit of the title compound, Fig. 1, comprises two crystallographically independent molecules (A and B). The bond lengths in the complex are normal (Allen *et al.*, 1987). The geometry around the Ni^II^ atoms in each molecule is distorted square planar. The dihedral angles between the coordination planes (O1—Ni1—N1 and O2—Ni1—N2 in molecule A and O3—Ni2—N3 and O4—Ni2—N4 in molecule B) are 13.43 (24) and 11.83 (32) Å, respectively. The dihedral angles between the two phenoxy rings (C1–C6) and (C24–C29) in molecule A, and (C32–C37) and (C55–C60) in molecule B, are 17.8 (4) and 36.5 (4)°, respectively.

The crystal packing is stabilized by weak π–π interactions [*Cg*1···*Cg*1^i^ = 3.758 (5) Å, perpendiculaire separation 3.750 (4) Å, slippage 1.171 Å; (i) 2 - x, 2 - y, 1 - z; *Cg*1 is the centroid of benzene ring (C1-C6)]. There are also a number of C-H···π interactions present (Table 1).

### Experimental

The title compound was synthesized by adding a methanolic solution (25 ml) of bis(2-hydroxybenzophenone)-2,2'-dimethyl propanediimine (2 mmol) to a solution of NiCl_2_.6H_2_O (2 mmol in 25 ml ethanol). The mixture was refluxed with stirring for 30 min. The resultant green solution was filtered. Dark-red single crystals, suitable for *X*-ray diffraction analysis, were obtained by recrystallization from ethanol by slow evaporation of the solvent at room temperature over several days.

### Refinement

The quality of the crystal was not optimal and it diffracted weakly; only 29% of the data can be considered to be observed [I>2σ(I)]. Although recrystallization was attempted repeatedly, better crystals could not be obtained. The C-bound H-atoms were included in calculated positions and treated as riding atoms: C-H = 0.93, 0.97 and 0.96 Å for CH, CH_2_ and CH_3_ H-atoms, respectively, with U_iso_(H) = k × U_eq_(parent C-atom), where k = 1.5 for CH_3_ H-atoms and k = 1.2 for all other H-atoms.

### Crystal data

**Table:**

[Ni(C_31_H_28_N_2_O_2_)]	*F*(000) = 2176
*M**_r_* = 519.26	*D*_x_ = 1.379 Mg m^−^^3^
Monoclinic, *P*2_1_/*c*	Mo *K*α radiation, λ = 0.71073 Å
Hall symbol: -P 2ybc	Cell parameters from 220 reflections
*a* = 23.722 (3) Å	θ = 2.9–20.0°
*b* = 9.4716 (6) Å	µ = 0.81 mm^−^^1^
*c* = 26.961 (4) Å	*T* = 291 K
β = 124.319 (9)°	Block, dark-red
*V* = 5003.2 (10) Å^3^	0.24 × 0.12 × 0.08 mm
*Z* = 8	

### Data collection

**Table:**

Stoe IPDS 2T image-plate diffractometer	8608 independent reflections
Radiation source: fine-focus sealed tube	2512 reflections with *I* > 2σ(*I*)
graphite	*R*_int_ = 0.117
ω scans	θ_max_ = 25.0°, θ_min_ = 1.8°
Absorption correction: multi-scan [*MULABS* (Blessing, 1995) in *PLATON* (Spek, 2009)]	*h* = −28→27
*T*_min_ = 0.830, *T*_max_ = 1.000	*k* = −10→11
23324 measured reflections	*l* = −30→32

### Refinement

**Table:**

Refinement on *F*^2^	Primary atom site location: structure-invariant direct methods
Least-squares matrix: full	Secondary atom site location: difference Fourier map
*R*[*F*^2^ > 2σ(*F*^2^)] = 0.053	Hydrogen site location: inferred from neighbouring sites
*wR*(*F*^2^) = 0.097	H-atom parameters constrained
*S* = 0.61	*w* = 1/[σ^2^(*F*_o_^2^) + (0.005*P*)^2^] where *P* = (*F*_o_^2^ + 2*F*_c_^2^)/3
8608 reflections	(Δ/σ)_max_ = 0.001
649 parameters	Δρ_max_ = 0.23 e Å^−^^3^
0 restraints	Δρ_min_ = −0.24 e Å^−^^3^

### Special details

**Table:**

Geometry. All e.s.d.'s (except the e.s.d. in the dihedral angle between two l.s. planes) are estimated using the full covariance matrix. The cell e.s.d.'s are taken into account individually in the estimation of e.s.d.'s in distances, angles and torsion angles; correlations between e.s.d.'s in cell parameters are only used when they are defined by crystal symmetry. An approximate (isotropic) treatment of cell e.s.d.'s is used for estimating e.s.d.'s involving l.s. planes.
Refinement. Refinement of *F*^2^ against ALL reflections. The weighted *R*-factor *wR* and goodness of fit *S* are based on *F*^2^, conventional *R*-factors *R* are based on *F*, with *F* set to zero for negative *F*^2^. The threshold expression of *F*^2^ > σ(*F*^2^) is used only for calculating *R*-factors(gt) *etc*. and is not relevant to the choice of reflections for refinement. *R*-factors based on *F*^2^ are statistically about twice as large as those based on *F*, and *R*- factors based on ALL data will be even larger.

### Fractional atomic coordinates and isotropic or equivalent isotropic displacement parameters (Å^2^)

**Table:**

	*x*	*y*	*z*	*U*_iso_*/*U*_eq_	
Ni1	1.02997 (5)	0.78653 (13)	0.65990 (4)	0.0311 (3)	
O1	1.0842 (2)	0.8006 (7)	0.6312 (2)	0.0507 (19)	
O2	1.1123 (2)	0.8077 (7)	0.7328 (2)	0.054 (2)	
N1	0.9521 (3)	0.7266 (8)	0.5836 (2)	0.0324 (19)	
N2	0.9807 (3)	0.8051 (7)	0.6956 (2)	0.0290 (18)	
C1	1.0658 (4)	0.8149 (10)	0.5766 (3)	0.034 (2)	
C2	1.1152 (4)	0.8582 (9)	0.5660 (3)	0.038 (3)	
H2A	1.1595	0.8755	0.5989	0.046*	
C3	1.1012 (4)	0.8759 (10)	0.5096 (3)	0.044 (3)	
H3A	1.1352	0.9040	0.5046	0.052*	
C4	1.0358 (4)	0.8513 (10)	0.4605 (3)	0.047 (3)	
H4A	1.0253	0.8636	0.4220	0.057*	
C5	0.9863 (4)	0.8089 (10)	0.4683 (3)	0.043 (3)	
H5A	0.9424	0.7934	0.4346	0.052*	
C6	0.9993 (3)	0.7877 (9)	0.5257 (3)	0.031 (2)	
C7	0.9468 (3)	0.7330 (9)	0.5322 (3)	0.033 (2)	
C8	0.8828 (4)	0.6750 (10)	0.4753 (3)	0.030 (2)	
C9	0.8779 (4)	0.5353 (10)	0.4586 (3)	0.044 (3)	
H9A	0.9147	0.4745	0.4813	0.053*	
C10	0.8181 (4)	0.4868 (10)	0.4081 (3)	0.050 (3)	
H10A	0.8147	0.3923	0.3974	0.061*	
C11	0.7642 (4)	0.5735 (11)	0.3738 (3)	0.048 (3)	
H11A	0.7236	0.5378	0.3409	0.057*	
C12	0.7699 (4)	0.7168 (11)	0.3879 (3)	0.046 (3)	
H12A	0.7341	0.7778	0.3628	0.055*	
C13	0.8287 (4)	0.7687 (10)	0.4393 (3)	0.046 (3)	
H13A	0.8323	0.8635	0.4497	0.055*	
C14	0.9025 (3)	0.6505 (8)	0.5891 (3)	0.033 (2)	
H14A	0.9262	0.5774	0.6192	0.040*	
H14B	0.8704	0.6041	0.5511	0.040*	
C15	0.8626 (4)	0.7401 (9)	0.6057 (3)	0.034 (2)	
C16	0.9092 (3)	0.8557 (9)	0.6521 (3)	0.036 (2)	
H16A	0.9100	0.9374	0.6308	0.044*	
H16B	0.8900	0.8851	0.6741	0.044*	
C17	1.0024 (4)	0.7955 (9)	0.7519 (3)	0.028 (2)	
C18	0.9564 (4)	0.8038 (10)	0.7730 (3)	0.030 (2)	
C19	0.9331 (4)	0.6818 (10)	0.7838 (3)	0.040 (2)	
H19A	0.9454	0.5946	0.7768	0.048*	
C20	0.8916 (3)	0.6868 (11)	0.8048 (3)	0.042 (3)	
H20A	0.8759	0.6038	0.8116	0.051*	
C21	0.8737 (4)	0.8168 (14)	0.8157 (4)	0.056 (3)	
H21A	0.8464	0.8216	0.8304	0.068*	
C22	0.8962 (4)	0.9379 (11)	0.8048 (4)	0.051 (3)	
H22A	0.8833	1.0249	0.8113	0.061*	
C23	0.9380 (4)	0.9330 (10)	0.7842 (3)	0.038 (3)	
H23A	0.9538	1.0163	0.7778	0.046*	
C24	1.0757 (3)	0.7739 (9)	0.7995 (3)	0.032 (2)	
C25	1.0976 (4)	0.7541 (9)	0.8595 (3)	0.042 (3)	
H25A	1.0650	0.7507	0.8684	0.051*	
C26	1.1646 (4)	0.7396 (10)	0.9052 (3)	0.054 (3)	
H26A	1.1777	0.7251	0.9444	0.065*	
C27	1.2131 (4)	0.7473 (11)	0.8914 (3)	0.056 (3)	
H27A	1.2592	0.7380	0.9219	0.067*	
C28	1.1944 (4)	0.7678 (10)	0.8345 (3)	0.054 (3)	
H28A	1.2280	0.7718	0.8269	0.065*	
C29	1.1253 (4)	0.7834 (10)	0.7860 (3)	0.035 (2)	
C30	0.8047 (3)	0.8248 (9)	0.5513 (3)	0.055 (3)	
H30A	0.7736	0.7605	0.5201	0.083*	
H30B	0.8240	0.8866	0.5363	0.083*	
H30C	0.7807	0.8797	0.5637	0.083*	
C31	0.8321 (4)	0.6435 (10)	0.6292 (3)	0.059 (3)	
H31A	0.8679	0.5918	0.6631	0.088*	
H31B	0.8013	0.5786	0.5982	0.088*	
H31C	0.8077	0.6986	0.6411	0.088*	
Ni2	0.53222 (5)	0.89266 (12)	0.72050 (4)	0.0273 (3)	
O3	0.6131 (2)	0.9183 (7)	0.7276 (2)	0.0426 (18)	
O4	0.5829 (2)	0.9419 (7)	0.8019 (2)	0.0405 (18)	
N3	0.4869 (3)	0.8180 (7)	0.6412 (2)	0.0281 (18)	
N4	0.4505 (3)	0.9045 (7)	0.7181 (2)	0.0285 (18)	
C32	0.6266 (4)	0.9007 (10)	0.6874 (3)	0.032 (2)	
C33	0.6938 (4)	0.9313 (9)	0.7043 (3)	0.038 (2)	
H33A	0.7255	0.9647	0.7429	0.046*	
C34	0.7135 (4)	0.9136 (10)	0.6662 (3)	0.045 (3)	
H34A	0.7576	0.9371	0.6782	0.054*	
C35	0.6674 (4)	0.8602 (9)	0.6091 (3)	0.046 (3)	
H35A	0.6811	0.8439	0.5833	0.056*	
C36	0.6019 (4)	0.8316 (9)	0.5906 (3)	0.037 (3)	
H36A	0.5714	0.7994	0.5516	0.044*	
C37	0.5787 (3)	0.8485 (9)	0.6275 (3)	0.028 (2)	
C38	0.5097 (3)	0.8071 (9)	0.6077 (3)	0.025 (2)	
C39	0.4662 (4)	0.7477 (10)	0.5448 (3)	0.034 (2)	
C40	0.4368 (4)	0.8401 (10)	0.4967 (3)	0.043 (3)	
H40A	0.4425	0.9370	0.5033	0.051*	
C41	0.3986 (4)	0.7873 (13)	0.4380 (3)	0.053 (3)	
H41A	0.3800	0.8483	0.4053	0.063*	
C42	0.3889 (4)	0.6428 (13)	0.4292 (4)	0.054 (3)	
H42A	0.3618	0.6070	0.3904	0.065*	
C43	0.4190 (4)	0.5516 (11)	0.4773 (4)	0.054 (3)	
H43A	0.4135	0.4547	0.4710	0.065*	
C44	0.4577 (4)	0.6055 (10)	0.5355 (3)	0.043 (2)	
H44A	0.4778	0.5443	0.5681	0.051*	
C45	0.4199 (3)	0.7514 (8)	0.6211 (3)	0.029 (2)	
H45A	0.4272	0.6851	0.6516	0.035*	
H45B	0.4036	0.6983	0.5846	0.035*	
C46	0.3645 (3)	0.8560 (9)	0.6092 (3)	0.033 (2)	
C47	0.3940 (3)	0.9652 (9)	0.6615 (3)	0.036 (2)	
H47A	0.4099	1.0480	0.6517	0.043*	
H47B	0.3581	0.9947	0.6663	0.043*	
C48	0.4448 (3)	0.8768 (9)	0.7615 (3)	0.026 (2)	
C49	0.3789 (3)	0.8908 (11)	0.7567 (3)	0.029 (2)	
C50	0.3352 (4)	0.7777 (11)	0.7428 (3)	0.044 (3)	
H50A	0.3467	0.6880	0.7372	0.053*	
C51	0.2742 (4)	0.8000 (12)	0.7373 (3)	0.056 (3)	
H51A	0.2445	0.7248	0.7278	0.067*	
C52	0.2570 (4)	0.9323 (12)	0.7456 (4)	0.058 (3)	
H52A	0.2154	0.9462	0.7409	0.070*	
C53	0.2999 (4)	1.0415 (11)	0.7606 (4)	0.055 (3)	
H53A	0.2882	1.1298	0.7673	0.066*	
C54	0.3611 (4)	1.0242 (10)	0.7661 (4)	0.044 (3)	
H54A	0.3903	1.1006	0.7760	0.052*	
C55	0.5051 (4)	0.8366 (9)	0.8212 (3)	0.031 (2)	
C56	0.4964 (3)	0.7722 (10)	0.8633 (3)	0.041 (3)	
H56A	0.4532	0.7419	0.8516	0.049*	
C57	0.5511 (4)	0.7536 (10)	0.9217 (3)	0.051 (3)	
H57A	0.5453	0.7076	0.9491	0.061*	
C58	0.6140 (4)	0.8031 (11)	0.9394 (3)	0.052 (3)	
H58A	0.6503	0.7936	0.9794	0.063*	
C59	0.6246 (4)	0.8659 (10)	0.8999 (3)	0.050 (3)	
H59A	0.6679	0.8988	0.9134	0.060*	
C60	0.5695 (4)	0.8823 (10)	0.8375 (3)	0.029 (2)	
C61	0.3381 (4)	0.9449 (10)	0.5511 (3)	0.059 (3)	
H61A	0.3197	0.8827	0.5172	0.088*	
H61B	0.3752	0.9980	0.5556	0.088*	
H61C	0.3031	1.0084	0.5448	0.088*	
C62	0.3060 (3)	0.7700 (10)	0.6021 (4)	0.058 (3)	
H62A	0.3223	0.7170	0.6381	0.087*	
H62B	0.2886	0.7063	0.5688	0.087*	
H62C	0.2702	0.8325	0.5948	0.087*	

### Atomic displacement parameters (Å^2^)

**Table:**

	*U*^11^	*U*^22^	*U*^33^	*U*^12^	*U*^13^	*U*^23^
Ni1	0.0228 (5)	0.0430 (8)	0.0227 (5)	−0.0028 (6)	0.0098 (4)	0.0008 (6)
O1	0.026 (3)	0.097 (6)	0.028 (3)	−0.009 (3)	0.015 (3)	0.001 (3)
O2	0.022 (3)	0.103 (6)	0.029 (3)	−0.010 (3)	0.010 (3)	−0.001 (3)
N1	0.030 (4)	0.043 (6)	0.027 (3)	−0.005 (4)	0.019 (3)	−0.001 (4)
N2	0.021 (3)	0.037 (5)	0.028 (3)	0.000 (3)	0.013 (3)	0.000 (3)
C1	0.039 (5)	0.038 (7)	0.037 (5)	0.003 (5)	0.028 (4)	−0.003 (4)
C2	0.028 (4)	0.053 (8)	0.037 (4)	−0.007 (4)	0.020 (4)	0.000 (4)
C3	0.042 (5)	0.059 (8)	0.037 (5)	−0.006 (5)	0.027 (4)	−0.007 (5)
C4	0.048 (5)	0.071 (9)	0.036 (5)	0.000 (5)	0.031 (5)	0.001 (5)
C5	0.032 (4)	0.062 (8)	0.026 (4)	−0.009 (5)	0.010 (4)	−0.004 (5)
C6	0.022 (4)	0.043 (6)	0.030 (4)	−0.009 (4)	0.017 (4)	0.001 (4)
C7	0.026 (4)	0.032 (6)	0.025 (4)	0.006 (4)	0.005 (4)	0.001 (4)
C8	0.028 (4)	0.035 (7)	0.024 (4)	−0.004 (4)	0.014 (4)	−0.005 (4)
C9	0.033 (5)	0.047 (7)	0.042 (5)	0.007 (5)	0.016 (4)	0.002 (5)
C10	0.048 (6)	0.045 (7)	0.039 (5)	−0.002 (5)	0.013 (5)	−0.011 (5)
C11	0.029 (5)	0.074 (9)	0.029 (4)	−0.026 (5)	0.009 (4)	−0.020 (5)
C12	0.031 (4)	0.072 (8)	0.030 (4)	0.011 (5)	0.014 (4)	0.007 (5)
C13	0.051 (5)	0.052 (7)	0.032 (4)	0.000 (5)	0.022 (4)	−0.007 (5)
C14	0.022 (4)	0.043 (7)	0.027 (4)	−0.005 (4)	0.009 (4)	0.003 (4)
C15	0.031 (4)	0.039 (7)	0.026 (4)	0.002 (4)	0.012 (4)	0.010 (4)
C16	0.030 (4)	0.044 (7)	0.034 (4)	0.015 (4)	0.018 (4)	0.015 (4)
C17	0.035 (4)	0.030 (6)	0.026 (4)	−0.003 (5)	0.022 (4)	0.004 (4)
C18	0.028 (4)	0.035 (6)	0.027 (4)	−0.005 (5)	0.016 (4)	−0.003 (5)
C19	0.036 (5)	0.036 (7)	0.044 (5)	−0.004 (4)	0.020 (4)	−0.010 (5)
C20	0.027 (4)	0.078 (9)	0.032 (4)	−0.012 (5)	0.023 (4)	0.004 (5)
C21	0.036 (5)	0.106 (11)	0.041 (5)	0.014 (6)	0.030 (4)	0.000 (6)
C22	0.041 (5)	0.053 (9)	0.057 (6)	0.006 (5)	0.026 (5)	0.001 (6)
C23	0.038 (5)	0.049 (8)	0.039 (5)	−0.009 (5)	0.029 (4)	−0.006 (5)
C24	0.016 (4)	0.046 (7)	0.026 (4)	0.002 (4)	0.006 (3)	0.006 (4)
C25	0.039 (5)	0.049 (8)	0.043 (5)	0.003 (5)	0.026 (4)	0.013 (5)
C26	0.038 (5)	0.093 (10)	0.017 (4)	0.002 (5)	0.006 (4)	0.019 (5)
C27	0.028 (4)	0.100 (10)	0.031 (4)	0.010 (5)	0.012 (4)	0.029 (5)
C28	0.029 (4)	0.093 (9)	0.041 (5)	0.000 (5)	0.020 (4)	0.012 (6)
C29	0.025 (4)	0.045 (7)	0.029 (4)	−0.007 (4)	0.011 (4)	−0.001 (5)
C30	0.034 (5)	0.081 (9)	0.039 (4)	0.011 (5)	0.014 (4)	0.010 (5)
C31	0.052 (5)	0.079 (9)	0.056 (5)	−0.023 (5)	0.037 (5)	−0.014 (5)
Ni2	0.0227 (5)	0.0371 (8)	0.0204 (5)	−0.0030 (6)	0.0112 (4)	−0.0031 (6)
O3	0.029 (3)	0.065 (5)	0.032 (3)	−0.003 (3)	0.016 (3)	0.002 (3)
O4	0.030 (3)	0.066 (5)	0.029 (3)	−0.016 (3)	0.018 (3)	−0.012 (3)
N3	0.023 (3)	0.026 (5)	0.035 (3)	0.001 (3)	0.016 (3)	−0.002 (3)
N4	0.024 (3)	0.034 (5)	0.021 (3)	−0.001 (3)	0.009 (3)	−0.004 (4)
C32	0.025 (4)	0.037 (6)	0.037 (5)	0.001 (4)	0.019 (4)	0.002 (5)
C33	0.032 (5)	0.042 (7)	0.032 (4)	−0.008 (4)	0.013 (4)	0.004 (4)
C34	0.039 (5)	0.066 (8)	0.042 (5)	−0.005 (5)	0.031 (4)	−0.008 (5)
C35	0.051 (5)	0.066 (8)	0.048 (5)	−0.002 (5)	0.044 (5)	−0.016 (5)
C36	0.031 (5)	0.053 (8)	0.021 (4)	−0.009 (4)	0.012 (4)	−0.006 (4)
C37	0.018 (4)	0.039 (7)	0.028 (4)	−0.001 (4)	0.014 (4)	−0.002 (4)
C38	0.033 (4)	0.017 (6)	0.032 (4)	0.006 (4)	0.022 (4)	0.010 (4)
C39	0.031 (4)	0.044 (7)	0.026 (4)	−0.005 (4)	0.015 (4)	−0.007 (4)
C40	0.039 (5)	0.051 (8)	0.033 (5)	−0.015 (5)	0.017 (4)	−0.005 (5)
C41	0.042 (5)	0.089 (9)	0.025 (4)	0.011 (6)	0.017 (4)	0.000 (6)
C42	0.049 (6)	0.088 (10)	0.019 (4)	−0.011 (6)	0.015 (4)	−0.028 (6)
C43	0.063 (6)	0.051 (8)	0.051 (6)	0.003 (6)	0.034 (5)	−0.012 (6)
C44	0.045 (5)	0.034 (7)	0.042 (5)	−0.002 (5)	0.021 (4)	−0.006 (5)
C45	0.031 (4)	0.027 (6)	0.025 (4)	−0.014 (4)	0.013 (3)	−0.005 (4)
C46	0.022 (4)	0.042 (7)	0.029 (4)	−0.003 (4)	0.010 (4)	0.003 (4)
C47	0.026 (4)	0.047 (7)	0.034 (4)	0.005 (4)	0.017 (4)	0.016 (4)
C48	0.022 (4)	0.021 (6)	0.032 (4)	0.005 (4)	0.015 (4)	0.007 (4)
C49	0.018 (4)	0.041 (6)	0.026 (4)	0.018 (5)	0.012 (3)	0.015 (5)
C50	0.039 (5)	0.050 (7)	0.046 (5)	−0.003 (5)	0.025 (4)	−0.005 (5)
C51	0.027 (5)	0.081 (9)	0.054 (5)	−0.001 (6)	0.021 (4)	0.029 (6)
C52	0.031 (5)	0.079 (10)	0.072 (7)	0.022 (6)	0.033 (5)	0.027 (7)
C53	0.048 (6)	0.041 (8)	0.073 (6)	0.012 (5)	0.033 (5)	0.001 (6)
C54	0.041 (5)	0.038 (7)	0.060 (6)	−0.006 (5)	0.033 (5)	−0.003 (5)
C55	0.034 (5)	0.043 (7)	0.023 (4)	0.004 (4)	0.020 (4)	0.005 (4)
C56	0.025 (4)	0.065 (8)	0.036 (4)	0.010 (5)	0.019 (4)	0.005 (5)
C57	0.056 (6)	0.065 (9)	0.041 (5)	0.021 (6)	0.032 (5)	0.017 (5)
C58	0.043 (5)	0.080 (9)	0.034 (5)	0.019 (6)	0.022 (4)	0.012 (6)
C59	0.025 (4)	0.082 (9)	0.043 (5)	0.008 (5)	0.020 (4)	−0.008 (5)
C60	0.028 (5)	0.034 (6)	0.032 (4)	0.005 (4)	0.020 (4)	−0.001 (4)
C61	0.055 (6)	0.066 (8)	0.041 (5)	0.009 (5)	0.019 (5)	0.007 (5)
C62	0.036 (5)	0.070 (9)	0.074 (6)	−0.012 (5)	0.035 (5)	−0.015 (6)

### Geometric parameters (Å, °)

**Table:**

Ni1—O2	1.841 (4)	Ni2—O3	1.833 (5)
Ni1—O1	1.841 (5)	Ni2—O4	1.872 (5)
Ni1—N2	1.896 (6)	Ni2—N4	1.904 (6)
Ni1—N1	1.918 (5)	Ni2—N3	1.907 (6)
O1—C1	1.286 (8)	O3—C32	1.304 (9)
O2—C29	1.307 (9)	O4—C60	1.298 (9)
N1—C7	1.319 (9)	N3—C38	1.292 (9)
N1—C14	1.458 (9)	N3—C45	1.498 (8)
N2—C17	1.302 (8)	N4—C48	1.281 (9)
N2—C16	1.497 (8)	N4—C47	1.466 (8)
C1—C6	1.413 (9)	C32—C33	1.419 (10)
C1—C2	1.415 (11)	C32—C37	1.441 (9)
C2—C3	1.369 (9)	C33—C34	1.358 (10)
C2—H2A	0.9300	C33—H33A	0.9300
C3—C4	1.378 (9)	C34—C35	1.387 (9)
C3—H3A	0.9300	C34—H34A	0.9300
C4—C5	1.363 (11)	C35—C36	1.365 (10)
C4—H4A	0.9300	C35—H35A	0.9300
C5—C6	1.410 (10)	C36—C37	1.391 (10)
C5—H5A	0.9300	C36—H36A	0.9300
C6—C7	1.449 (10)	C37—C38	1.460 (9)
C7—C8	1.528 (9)	C38—C39	1.511 (9)
C8—C9	1.381 (10)	C39—C44	1.364 (11)
C8—C13	1.403 (10)	C39—C40	1.384 (10)
C9—C10	1.377 (10)	C40—C41	1.400 (10)
C9—H9A	0.9300	C40—H40A	0.9300
C10—C11	1.354 (10)	C41—C42	1.386 (12)
C10—H10A	0.9300	C41—H41A	0.9300
C11—C12	1.395 (12)	C42—C43	1.375 (12)
C11—H11A	0.9300	C42—H42A	0.9300
C12—C13	1.390 (9)	C43—C44	1.393 (10)
C12—H12A	0.9300	C43—H43A	0.9300
C13—H13A	0.9300	C44—H44A	0.9300
C14—C15	1.514 (10)	C45—C46	1.528 (10)
C14—H14A	0.9700	C45—H45A	0.9700
C14—H14B	0.9700	C45—H45B	0.9700
C15—C31	1.510 (10)	C46—C62	1.527 (10)
C15—C30	1.553 (9)	C46—C47	1.562 (10)
C15—C16	1.557 (10)	C46—C61	1.564 (10)
C16—H16A	0.9700	C47—H47A	0.9700
C16—H16B	0.9700	C47—H47B	0.9700
C17—C24	1.481 (9)	C48—C55	1.477 (9)
C17—C18	1.490 (11)	C48—C49	1.500 (10)
C18—C19	1.381 (11)	C49—C50	1.387 (11)
C18—C23	1.388 (11)	C49—C54	1.399 (11)
C19—C20	1.386 (10)	C50—C51	1.385 (11)
C19—H19A	0.9300	C50—H50A	0.9300
C20—C21	1.388 (12)	C51—C52	1.374 (12)
C20—H20A	0.9300	C51—H51A	0.9300
C21—C22	1.366 (12)	C52—C53	1.343 (12)
C21—H21A	0.9300	C52—H52A	0.9300
C22—C23	1.384 (11)	C53—C54	1.385 (11)
C22—H22A	0.9300	C53—H53A	0.9300
C23—H23A	0.9300	C54—H54A	0.9300
C24—C25	1.405 (9)	C55—C60	1.400 (10)
C24—C29	1.415 (11)	C55—C56	1.403 (10)
C25—C26	1.361 (9)	C56—C57	1.375 (8)
C25—H25A	0.9300	C56—H56A	0.9300
C26—C27	1.398 (11)	C57—C58	1.367 (11)
C26—H26A	0.9300	C57—H57A	0.9300
C27—C28	1.347 (10)	C58—C59	1.362 (11)
C27—H27A	0.9300	C58—H58A	0.9300
C28—C29	1.415 (9)	C59—C60	1.444 (9)
C28—H28A	0.9300	C59—H59A	0.9300
C30—H30A	0.9600	C61—H61A	0.9600
C30—H30B	0.9600	C61—H61B	0.9600
C30—H30C	0.9600	C61—H61C	0.9600
C31—H31A	0.9600	C62—H62A	0.9600
C31—H31B	0.9600	C62—H62B	0.9600
C31—H31C	0.9600	C62—H62C	0.9600
			
O2—Ni1—O1	82.5 (2)	O3—Ni2—O4	84.3 (2)
O2—Ni1—N2	92.0 (2)	O3—Ni2—N4	168.5 (3)
O1—Ni1—N2	169.5 (3)	O4—Ni2—N4	89.7 (2)
O2—Ni1—N1	167.8 (3)	O3—Ni2—N3	93.6 (3)
O1—Ni1—N1	92.8 (2)	O4—Ni2—N3	172.1 (3)
N2—Ni1—N1	94.3 (2)	N4—Ni2—N3	93.6 (2)
C1—O1—Ni1	128.5 (5)	C32—O3—Ni2	129.0 (5)
C29—O2—Ni1	127.2 (5)	C60—O4—Ni2	119.7 (5)
C7—N1—C14	121.6 (6)	C38—N3—C45	119.4 (6)
C7—N1—Ni1	125.2 (5)	C38—N3—Ni2	128.2 (5)
C14—N1—Ni1	112.5 (5)	C45—N3—Ni2	112.0 (5)
C17—N2—C16	117.8 (6)	C48—N4—C47	121.6 (6)
C17—N2—Ni1	129.3 (5)	C48—N4—Ni2	126.0 (5)
C16—N2—Ni1	112.4 (4)	C47—N4—Ni2	112.1 (5)
O1—C1—C6	124.4 (8)	O3—C32—C33	117.8 (7)
O1—C1—C2	118.5 (7)	O3—C32—C37	124.6 (7)
C6—C1—C2	117.1 (7)	C33—C32—C37	117.6 (8)
C3—C2—C1	123.2 (7)	C34—C33—C32	122.4 (7)
C3—C2—H2A	118.4	C34—C33—H33A	118.8
C1—C2—H2A	118.4	C32—C33—H33A	118.8
C2—C3—C4	118.9 (8)	C33—C34—C35	119.4 (7)
C2—C3—H3A	120.6	C33—C34—H34A	120.3
C4—C3—H3A	120.6	C35—C34—H34A	120.3
C5—C4—C3	120.2 (7)	C36—C35—C34	120.1 (7)
C5—C4—H4A	119.9	C36—C35—H35A	119.9
C3—C4—H4A	119.9	C34—C35—H35A	119.9
C4—C5—C6	122.4 (7)	C35—C36—C37	122.9 (7)
C4—C5—H5A	118.8	C35—C36—H36A	118.5
C6—C5—H5A	118.8	C37—C36—H36A	118.5
C5—C6—C1	118.2 (7)	C36—C37—C32	117.5 (6)
C5—C6—C7	120.9 (6)	C36—C37—C38	121.4 (6)
C1—C6—C7	120.8 (6)	C32—C37—C38	121.0 (7)
N1—C7—C6	124.3 (6)	N3—C38—C37	123.3 (7)
N1—C7—C8	119.4 (7)	N3—C38—C39	121.5 (6)
C6—C7—C8	116.2 (6)	C37—C38—C39	115.2 (7)
C9—C8—C13	120.3 (7)	C44—C39—C40	120.4 (7)
C9—C8—C7	121.7 (7)	C44—C39—C38	120.8 (7)
C13—C8—C7	118.0 (8)	C40—C39—C38	118.7 (8)
C10—C9—C8	119.5 (8)	C39—C40—C41	119.8 (9)
C10—C9—H9A	120.3	C39—C40—H40A	120.1
C8—C9—H9A	120.3	C41—C40—H40A	120.1
C11—C10—C9	121.5 (9)	C42—C41—C40	119.0 (9)
C11—C10—H10A	119.2	C42—C41—H41A	120.5
C9—C10—H10A	119.2	C40—C41—H41A	120.5
C10—C11—C12	119.7 (7)	C43—C42—C41	120.8 (8)
C10—C11—H11A	120.2	C43—C42—H42A	119.6
C12—C11—H11A	120.2	C41—C42—H42A	119.6
C13—C12—C11	120.3 (8)	C42—C43—C44	119.6 (10)
C13—C12—H12A	119.9	C42—C43—H43A	120.2
C11—C12—H12A	119.9	C44—C43—H43A	120.2
C12—C13—C8	118.6 (9)	C39—C44—C43	120.4 (9)
C12—C13—H13A	120.7	C39—C44—H44A	119.8
C8—C13—H13A	120.7	C43—C44—H44A	119.8
N1—C14—C15	115.3 (7)	N3—C45—C46	114.5 (6)
N1—C14—H14A	108.5	N3—C45—H45A	108.6
C15—C14—H14A	108.5	C46—C45—H45A	108.6
N1—C14—H14B	108.5	N3—C45—H45B	108.6
C15—C14—H14B	108.5	C46—C45—H45B	108.6
H14A—C14—H14B	107.5	H45A—C45—H45B	107.6
C31—C15—C14	108.1 (7)	C62—C46—C45	107.1 (6)
C31—C15—C30	109.3 (6)	C62—C46—C47	112.2 (7)
C14—C15—C30	112.2 (6)	C45—C46—C47	110.2 (5)
C31—C15—C16	112.4 (6)	C62—C46—C61	109.8 (6)
C14—C15—C16	110.7 (6)	C45—C46—C61	111.8 (7)
C30—C15—C16	104.2 (7)	C47—C46—C61	105.8 (7)
N2—C16—C15	112.4 (6)	N4—C47—C46	111.2 (6)
N2—C16—H16A	109.1	N4—C47—H47A	109.4
C15—C16—H16A	109.1	C46—C47—H47A	109.4
N2—C16—H16B	109.1	N4—C47—H47B	109.4
C15—C16—H16B	109.1	C46—C47—H47B	109.4
H16A—C16—H16B	107.9	H47A—C47—H47B	108.0
N2—C17—C24	121.3 (7)	N4—C48—C55	120.8 (6)
N2—C17—C18	123.2 (6)	N4—C48—C49	123.1 (6)
C24—C17—C18	115.6 (6)	C55—C48—C49	116.0 (7)
C19—C18—C23	118.7 (8)	C50—C49—C54	119.5 (7)
C19—C18—C17	120.2 (9)	C50—C49—C48	122.6 (9)
C23—C18—C17	121.1 (9)	C54—C49—C48	118.0 (8)
C18—C19—C20	121.3 (9)	C51—C50—C49	119.1 (9)
C18—C19—H19A	119.4	C51—C50—H50A	120.4
C20—C19—H19A	119.4	C49—C50—H50A	120.4
C19—C20—C21	119.3 (9)	C52—C51—C50	120.6 (10)
C19—C20—H20A	120.3	C52—C51—H51A	119.7
C21—C20—H20A	120.3	C50—C51—H51A	119.7
C22—C21—C20	119.7 (9)	C53—C52—C51	120.5 (9)
C22—C21—H21A	120.2	C53—C52—H52A	119.7
C20—C21—H21A	120.2	C51—C52—H52A	119.7
C21—C22—C23	121.0 (10)	C52—C53—C54	120.8 (10)
C21—C22—H22A	119.5	C52—C53—H53A	119.6
C23—C22—H22A	119.5	C54—C53—H53A	119.6
C22—C23—C18	120.0 (10)	C53—C54—C49	119.4 (9)
C22—C23—H23A	120.0	C53—C54—H54A	120.3
C18—C23—H23A	120.0	C49—C54—H54A	120.3
C25—C24—C29	118.9 (6)	C60—C55—C56	120.9 (7)
C25—C24—C17	120.5 (7)	C60—C55—C48	118.5 (7)
C29—C24—C17	120.5 (6)	C56—C55—C48	120.0 (7)
C26—C25—C24	122.7 (8)	C57—C56—C55	120.5 (7)
C26—C25—H25A	118.7	C57—C56—H56A	119.7
C24—C25—H25A	118.7	C55—C56—H56A	119.7
C25—C26—C27	118.1 (7)	C58—C57—C56	119.7 (9)
C25—C26—H26A	121.0	C58—C57—H57A	120.1
C27—C26—H26A	121.0	C56—C57—H57A	120.1
C28—C27—C26	121.2 (7)	C59—C58—C57	121.6 (7)
C28—C27—H27A	119.4	C59—C58—H58A	119.2
C26—C27—H27A	119.4	C57—C58—H58A	119.2
C27—C28—C29	122.2 (8)	C58—C59—C60	120.9 (8)
C27—C28—H28A	118.9	C58—C59—H59A	119.6
C29—C28—H28A	118.9	C60—C59—H59A	119.6
O2—C29—C28	117.7 (7)	O4—C60—C55	125.5 (6)
O2—C29—C24	125.3 (6)	O4—C60—C59	118.1 (7)
C28—C29—C24	117.0 (7)	C55—C60—C59	116.3 (8)
C15—C30—H30A	109.5	C46—C61—H61A	109.5
C15—C30—H30B	109.5	C46—C61—H61B	109.5
H30A—C30—H30B	109.5	H61A—C61—H61B	109.5
C15—C30—H30C	109.5	C46—C61—H61C	109.5
H30A—C30—H30C	109.5	H61A—C61—H61C	109.5
H30B—C30—H30C	109.5	H61B—C61—H61C	109.5
C15—C31—H31A	109.5	C46—C62—H62A	109.5
C15—C31—H31B	109.5	C46—C62—H62B	109.5
H31A—C31—H31B	109.5	H62A—C62—H62B	109.5
C15—C31—H31C	109.5	C46—C62—H62C	109.5
H31A—C31—H31C	109.5	H62A—C62—H62C	109.5
H31B—C31—H31C	109.5	H62B—C62—H62C	109.5
			
O2—Ni1—O1—C1	−169.8 (8)	C17—C24—C29—C28	177.2 (8)
N2—Ni1—O1—C1	−111.3 (13)	O4—Ni2—O3—C32	177.9 (8)
N1—Ni1—O1—C1	21.5 (8)	N4—Ni2—O3—C32	−123.1 (13)
O1—Ni1—O2—C29	−165.6 (8)	N3—Ni2—O3—C32	5.5 (8)
N2—Ni1—O2—C29	23.3 (8)	O3—Ni2—O4—C60	−144.8 (6)
N1—Ni1—O2—C29	−97.8 (15)	N4—Ni2—O4—C60	45.0 (6)
O2—Ni1—N1—C7	−79.4 (17)	O3—Ni2—N3—C38	−5.1 (8)
O1—Ni1—N1—C7	−12.6 (8)	N4—Ni2—N3—C38	165.9 (7)
N2—Ni1—N1—C7	159.7 (8)	O3—Ni2—N3—C45	167.7 (5)
O2—Ni1—N1—C14	91.0 (14)	N4—Ni2—N3—C45	−21.3 (5)
O1—Ni1—N1—C14	157.9 (6)	O3—Ni2—N4—C48	−90.3 (15)
N2—Ni1—N1—C14	−29.9 (6)	O4—Ni2—N4—C48	−31.7 (8)
O2—Ni1—N2—C17	−16.1 (8)	N3—Ni2—N4—C48	141.1 (8)
O1—Ni1—N2—C17	−73.8 (17)	O3—Ni2—N4—C47	83.4 (13)
N1—Ni1—N2—C17	153.5 (8)	O4—Ni2—N4—C47	142.0 (5)
O2—Ni1—N2—C16	155.5 (5)	N3—Ni2—N4—C47	−45.2 (6)
O1—Ni1—N2—C16	97.7 (13)	Ni2—O3—C32—C33	178.6 (6)
N1—Ni1—N2—C16	−35.0 (6)	Ni2—O3—C32—C37	−3.3 (14)
Ni1—O1—C1—C6	−16.7 (14)	O3—C32—C33—C34	178.6 (9)
Ni1—O1—C1—C2	164.6 (6)	C37—C32—C33—C34	0.3 (14)
O1—C1—C2—C3	179.6 (9)	C32—C33—C34—C35	−1.8 (15)
C6—C1—C2—C3	0.8 (14)	C33—C34—C35—C36	2.8 (15)
C1—C2—C3—C4	0.3 (15)	C34—C35—C36—C37	−2.5 (15)
C2—C3—C4—C5	−0.6 (14)	C35—C36—C37—C32	1.0 (14)
C3—C4—C5—C6	−0.4 (15)	C35—C36—C37—C38	−175.4 (8)
C4—C5—C6—C1	1.5 (15)	O3—C32—C37—C36	−178.0 (9)
C4—C5—C6—C7	−175.6 (9)	C33—C32—C37—C36	0.1 (12)
O1—C1—C6—C5	179.6 (9)	O3—C32—C37—C38	−1.6 (14)
C2—C1—C6—C5	−1.7 (13)	C33—C32—C37—C38	176.6 (8)
O1—C1—C6—C7	−3.3 (14)	C45—N3—C38—C37	−170.0 (7)
C2—C1—C6—C7	175.4 (8)	Ni2—N3—C38—C37	2.3 (12)
C14—N1—C7—C6	−170.1 (7)	C45—N3—C38—C39	9.1 (12)
Ni1—N1—C7—C6	−0.5 (12)	Ni2—N3—C38—C39	−178.6 (5)
C14—N1—C7—C8	7.6 (12)	C36—C37—C38—N3	178.3 (8)
Ni1—N1—C7—C8	177.2 (6)	C32—C37—C38—N3	2.0 (13)
C5—C6—C7—N1	−171.3 (9)	C36—C37—C38—C39	−0.9 (12)
C1—C6—C7—N1	11.7 (13)	C32—C37—C38—C39	−177.1 (8)
C5—C6—C7—C8	11.0 (12)	N3—C38—C39—C44	−79.4 (11)
C1—C6—C7—C8	−166.0 (8)	C37—C38—C39—C44	99.8 (9)
N1—C7—C8—C9	−88.6 (11)	N3—C38—C39—C40	102.9 (10)
C6—C7—C8—C9	89.3 (10)	C37—C38—C39—C40	−78.0 (10)
N1—C7—C8—C13	92.5 (10)	C44—C39—C40—C41	−0.5 (13)
C6—C7—C8—C13	−89.6 (9)	C38—C39—C40—C41	177.3 (7)
C13—C8—C9—C10	−3.6 (14)	C39—C40—C41—C42	2.2 (13)
C7—C8—C9—C10	177.5 (7)	C40—C41—C42—C43	−3.1 (15)
C8—C9—C10—C11	1.1 (14)	C41—C42—C43—C44	2.2 (15)
C9—C10—C11—C12	3.0 (14)	C40—C39—C44—C43	−0.5 (14)
C10—C11—C12—C13	−4.5 (14)	C38—C39—C44—C43	−178.2 (8)
C11—C12—C13—C8	2.1 (12)	C42—C43—C44—C39	−0.4 (14)
C9—C8—C13—C12	2.0 (12)	C38—N3—C45—C46	−116.3 (8)
C7—C8—C13—C12	−179.1 (7)	Ni2—N3—C45—C46	70.2 (7)
C7—N1—C14—C15	−117.5 (8)	N3—C45—C46—C62	−168.5 (6)
Ni1—N1—C14—C15	71.7 (7)	N3—C45—C46—C47	−46.1 (8)
N1—C14—C15—C31	−161.6 (6)	N3—C45—C46—C61	71.2 (8)
N1—C14—C15—C30	77.8 (8)	C48—N4—C47—C46	−110.5 (9)
N1—C14—C15—C16	−38.1 (8)	Ni2—N4—C47—C46	75.5 (7)
C17—N2—C16—C15	−114.9 (8)	C62—C46—C47—N4	91.3 (8)
Ni1—N2—C16—C15	72.5 (7)	C45—C46—C47—N4	−28.0 (9)
C31—C15—C16—N2	85.4 (8)	C61—C46—C47—N4	−149.0 (7)
C14—C15—C16—N2	−35.6 (8)	C47—N4—C48—C55	−170.5 (7)
C30—C15—C16—N2	−156.4 (6)	Ni2—N4—C48—C55	2.6 (13)
C16—N2—C17—C24	−167.8 (7)	C47—N4—C48—C49	5.8 (14)
Ni1—N2—C17—C24	3.4 (13)	Ni2—N4—C48—C49	178.9 (7)
C16—N2—C17—C18	12.8 (13)	N4—C48—C49—C50	95.3 (11)
Ni1—N2—C17—C18	−176.0 (7)	C55—C48—C49—C50	−88.2 (10)
N2—C17—C18—C19	96.9 (10)	N4—C48—C49—C54	−84.0 (11)
C24—C17—C18—C19	−82.6 (9)	C55—C48—C49—C54	92.5 (9)
N2—C17—C18—C23	−85.4 (11)	C54—C49—C50—C51	1.3 (12)
C24—C17—C18—C23	95.2 (9)	C48—C49—C50—C51	−178.0 (7)
C23—C18—C19—C20	0.6 (11)	C49—C50—C51—C52	−0.2 (13)
C17—C18—C19—C20	178.4 (6)	C50—C51—C52—C53	−1.5 (14)
C18—C19—C20—C21	−0.5 (11)	C51—C52—C53—C54	2.0 (15)
C19—C20—C21—C22	0.9 (11)	C52—C53—C54—C49	−0.8 (14)
C20—C21—C22—C23	−1.4 (12)	C50—C49—C54—C53	−0.9 (12)
C21—C22—C23—C18	1.4 (12)	C48—C49—C54—C53	178.5 (7)
C19—C18—C23—C22	−1.0 (11)	N4—C48—C55—C60	25.5 (13)
C17—C18—C23—C22	−178.8 (7)	C49—C48—C55—C60	−151.1 (8)
N2—C17—C24—C25	−175.6 (9)	N4—C48—C55—C56	−163.6 (9)
C18—C17—C24—C25	3.9 (12)	C49—C48—C55—C56	19.8 (12)
N2—C17—C24—C29	9.1 (13)	C60—C55—C56—C57	0.5 (14)
C18—C17—C24—C29	−171.4 (9)	C48—C55—C56—C57	−170.2 (8)
C29—C24—C25—C26	−1.9 (14)	C55—C56—C57—C58	2.5 (14)
C17—C24—C25—C26	−177.2 (8)	C56—C57—C58—C59	−2.6 (16)
C24—C25—C26—C27	1.0 (15)	C57—C58—C59—C60	−0.2 (15)
C25—C26—C27—C28	−0.3 (17)	Ni2—O4—C60—C55	−32.1 (12)
C26—C27—C28—C29	0.3 (17)	Ni2—O4—C60—C59	150.6 (6)
Ni1—O2—C29—C28	162.6 (6)	C56—C55—C60—O4	179.5 (9)
Ni1—O2—C29—C24	−18.6 (15)	C48—C55—C60—O4	−9.7 (13)
C27—C28—C29—O2	177.7 (10)	C56—C55—C60—C59	−3.2 (13)
C27—C28—C29—C24	−1.1 (15)	C48—C55—C60—C59	167.6 (7)
C25—C24—C29—O2	−176.9 (9)	C58—C59—C60—O4	−179.4 (9)
C17—C24—C29—O2	−1.6 (15)	C58—C59—C60—C55	3.1 (13)
C25—C24—C29—C28	1.8 (14)		

### Table 1 Table 1. C—H···π interactions (Å, °)

Cg2, Cg3 and Cg4 are the centroids of the C8–C23, C32–C37 and C55–C60 rings, respectively.

**Table:**

C—H···Cg	C—H	H···Cg	C···Cg	C—H···Cg
C21-H21A···Cg2^ii^	0.93	2.90	3.757 (11)	153
C41-H41A···Cg3^iii^	0.93	2.83	3.680 (12)	153
C44-H44A···Cg4^iv^	0.93	2.95	3.708 (10)	149
C47-H47A···Cg4^v^	0.93	2.92	3.884 (9)	171

Symmetry codes: (ii) x, 3/2-y, 1/2+z; (iii) 1-x, 2-y, 1-z; (iv) 1-x, -1/2+y, 3/2-z; (v) 1-x, 1/2+y, 3/2-z.
